# SARS-CoV-2 nosocomial infection acquired in a French university hospital during the 1st wave of the Covid-19 pandemic, a prospective study

**DOI:** 10.1186/s13756-021-00984-x

**Published:** 2021-08-05

**Authors:** A. Landoas, F. Cazzorla, M. Gallouche, S. Larrat, B. Nemoz, C. Giner, M. Le Maréchal, P. Pavese, O. Epaulard, P. Morand, M.-R. Mallaret, C. Landelle

**Affiliations:** 1 Infection Control Unit, Grenoble Alpes University Hospital, Grenoble, France; 2 Grenoble Alpes University/CNRS, Grenoble INP, MESP TIM-C UMR 5525, Grenoble, France; 3 Virology Laboratory, Grenoble Alpes University Hospital, Grenoble, France; 4 Grenoble Alpes University/CNRS/CEA, Institut de Biologie Structurale (IBS), HIV and persistent viral infections, Grenoble, France; 5 Infectious Diseases Department, Grenoble Alpes University Hospital, Grenoble, France; 6 Hospital Hygiene Department, Pavilion E - Grenoble Alpes University Hospital, CS 10217, 38043 Grenoble Cedex 9, France

**Keywords:** SARS-CoV-2, COVID-19, Outbreak, Healthcare-associated infection, Mask, Hand hygiene

## Abstract

**Background:**

In healthcare facilities, nosocomial transmissions of respiratory viruses are a major issue. SARS-CoV-2 is not exempt from nosocomial transmission. Our goals were to describe COVID-19 nosocomial cases during the first pandemic wave among patients in a French university hospital and compliance with hygiene measures.

**Methods:**

We conducted a prospective observational study in Grenoble Alpes University Hospital from 01/03/2020 to 11/05/2020. We included all hospitalised patients with a documented SARS-CoV-2 diagnosis. Nosocomial case was defined by a delay of 5 days between hospitalisation and first symptoms. Hygiene measures were evaluated between 11/05/2020 and 22/05/2020. Lockdown measures were effective in France on 17/03/2020 and ended on 11/05/2020. Systematic wearing of mask was mandatory for all healthcare workers (HCW) and visits were prohibited in our institution from 13/03/2021 and for the duration of the lockdown period.

**Results:**

Among 259 patients included, 14 (5.4%) were considered as nosocomial COVID-19. Median time before symptom onset was 25 days (interquartile range: 12–42). Eleven patients (79%) had risk factors for severe COVID-19. Five died (36%) including 4 deaths attributable to COVID-19. Two clusters were identified. The first cluster had 5 cases including 3 nosocomial acquisitions and no tested HCWs were positive. The second cluster had 3 cases including 2 nosocomial cases and 4 HCWs were positive. Surgical mask wearing and hand hygiene compliance were adequate for 95% and 61% of HCWs, respectively.

**Conclusions:**

The number of nosocomial COVID-19 cases in our hospital was low. Compliance regarding mask wearing, hand hygiene and lockdown measures drastically reduced transmission of the virus. Monitoring of nosocomial COVID-19 cases during the first wave enabled us to determine to what extent the hygiene measures taken were effective and patients protected.

*Trial registration* Study ethics approval was obtained retrospectively on 30 September 2020 (CECIC Rhône-Alpes-Auvergne, Clermont-Ferrand, IRB 5891).

## Background

Severe Acute Respiratory Syndrome coronavirus 2 (SARS-CoV-2), responsible for Coronavirus disease 19 (COVID-19), rapidly spread all around the world and was declared a pandemic on 11/03/2020 by the World Health Organization (WHO) [1, 2]. Given that the elderly or persons with comorbidities are more likely to develop serious disease [3], preventing the acquisition of SARS-CoV-2 within healthcare facilities rapidly became a major challenge.

During the first wave, the French government chose to apply strict containment measures and massive lockdown from 17/03/2020 to 11/05/2020. Isolation and social distancing measures were established such as the closing of schools and public spaces and the prohibition of travel, and different barrier measures (washing hands regularly, coughing inside the elbow, etc.) were strongly recommended. Despite these measures, by 31/05/2020 in France, SARS-CoV-2 had resulted in almost 150,000 cases, more than 28,000 deaths and about 17,000 hospitalisations [4]. While SARS-CoV-2 is spread primarily through droplets, transmission by contact with contaminated objects and surfaces or aerosols can also occur [5]. A number of measures were taken to prevent nosocomial transmission, including: strict ban on hospital visits, strengthened hand hygiene, and systematic wearing of a surgical mask by healthcare workers (HCWs) [6].

In this study, we sought to describe nosocomial cases and clusters of COVID-19 cases acquired in a French university hospital during the first wave of the COVID-19 epidemic. A secondary aim was to assess compliance with hygiene measures implemented during the epidemic period.

## Methods

### Generalities

We conducted a prospective observational study during the first wave of the COVID-19 epidemic period in Grenoble Alpes University Hospital (CHU-GA), from 01/03/2020 to 11/05/2020. CHU-GA is the largest hospital of Grenoble city (France) with more than 9500 employees, over 2100 beds in 149 units (86 medical units, 47 surgery units and 16 intensive care units (ICU)/postICU) with 65% of double rooms; approximately 2400 patients are admitted every day.

### General measures at Grenoble Alpes University Hospital

During the epidemic period CHU-GA was designated as the COVID-19 reference hospital for the department of Isère. Overall organisation was modified so as to reduce normal hospital activity, including a ban on elective surgery for the duration of the lockdown period, the objective being to cope with a massive influx of COVID-19 patients. Surgical mask wearing within the hospital was mandatory for HCWs and for patients when moving outside of their room or during care. Units reserved for COVID-19 patients were set up, where droplets and contact precautions (surgical mask, gloves, gown) were required. FFP2 masks were also used, during aerosol-generating procedures and in accordance with national French recommendations [7]. Visits in our institution were prohibited from 13/03/2021 and for the duration of the lockdown period. A maximum of 157 beds were available in ICU and 186 in other COVID-19 dedicated units. When a case of COVID-19 was suspected in a non-COVID area, the patient was tested and transferred to a COVID area in case of positivity. CHU-GA hospitalised only seriously ill patients or those with comorbidities. All others remained home and followed up by regular phone calls in accordance with a special and dedicated protocol. The testing criteria were not only clinical symptoms commonly suggestive of COVID-19 such as fever, dry cough and tiredness, but also a suspicious computed tomography scan (CT-scan) [8]. All HCWs with suspected COVID-19 were tested as well. The first-line approach for testing was nasopharyngeal swab. In case of doubt or negative result with strong clinical suspicion, clinicians used imagery or clinical evidence to confirm or refute the assumption, which was possibly corroborated by a deeper sample such as tracheal or bronchoalveolar-lavage fluid. Samples were analysed in the virology laboratory by real-time reverse transcription polymerase chain reaction (RT-PCR) assay for SARS-CoV-2. Contact cases were defined as HCWs or patients with close contact (< 1 m, ≥ 15 min) to clinical cases without mask (roommates, shared activities etc.). Contact cases were monitored closely but tested only if symptomatic.

### The patients included

All hospitalised patients with positive RT-PCR results for SARS-CoV-2 or CT-scan signs suggestive or typical of COVID-19 (levels 4 and 5 of CO-RADS score) were included. Non-hospitalised patients (i.e. emergency stay, consultation, etc.), even those with a positive result (positive RT-PCR or radiologic evidence), were excluded. A COVID-19 case was considered as nosocomial if onset of symptoms occurred more than 5 days after hospitalisation, considering that 5 days is the median incubation time for COVID-19 [9]. Cases attributable to other healthcare facilities were not considered in our study as nosocomial cases. At that time there was no consensual nationwide definition for nosocomial clusters. We used the following definition: ≥ 2 nosocomial cases among patients and HCWs with fewer than 7 days between cases.

### Data collection

Patient data were collected from their electronic medical records by residents of the hospital hygiene unit. They included age, sex, hospitalisation unit, onset of symptoms, co-infection, risk factors for severe COVID-19 (overweight, obesity with Body Mass Index > 30, hypertension, diabetes, pulmonary disease, cardiac disease, neuromuscular disease, kidney disease, immunodeficiency), ICU stay, clinical evolution, origins of infection (community-acquired or nosocomial). For nosocomial cases, investigations were extensive, collecting information concerning the acquisition unit and patient room setting (double or single room) and all nosocomial contact generated (patients and HCWs). The study complied with the Outbreak Reports and Intervention studies Of Nosocomial infection (ORION) reporting guidelines [10].

Between 11/05/2020 and 22/05/2020, audits were carried out in all non-COVID acute care units (88 units) to assess compliance with hygiene measures. Observations were carried out by nurses from the hygiene unit and pertained to the following items: correct mask wearing by HCWs and patients when needed, hand hygiene, physical distancing, screen deployment in double occupancy rooms and disinfection of shared equipment.

### Analyses and ethical aspects

Qualitative variables were expressed in absolute number and in percentage. Quantitative variables were expressed as median and interquartile range (IQR). Groups were compared by means of Fisher’s exact test. Ethics approval was obtained on 30 September 2020 (CECIC Rhône-Alpes-Auvergne, Clermont-Ferrand, IRB 5891).

## Results

From 01/03/2020 to 11/05/2020, 4811 samples from suspected patients and HCWs were analysed in the virology laboratory in search of SARS-CoV-2, and 259 hospitalised patients with COVID-19 were identified. Among them, 62 (23.9%) were transferred to the ICU; 215 (83.0%) were discharged and 37 (14.3%) died. All in all, 245 (94.6%) cases were community-acquired and 14 (5.4%) were considered as nosocomial COVID-19 cases.

Concerning nosocomial COVID-19 patients, 8 (57.0%) were male; median age was 63.7 years (IQR: 45.8–83.3) (Table 1). Median time between hospitalisation and symptom onset was 24.5 days (IQR: 11.5–42), four patients had symptoms between 5 and 14 days after hospitalization. Eleven (78.6%) had risk factors other than age exceeding 65 years. In this population, 2 (14.3%) patients were overweight, but neither was clinically obese. However, half of them had a past history of pulmonary disease such as asthma or chronic obstructive pulmonary disease, 6 (42.9%) had hypertension, 5 (35.7%) had cardiovascular disease and 4 (28.6%) presented with diabetes. Five of them (35.7%) died. In comparison with community cases, mortality among nosocomial cases was higher (13.1% vs 35.7%; p < 0.005); in 4 out of the 5 (80%) cases, it was attributable to COVID-19. As regards the 14 nosocomial cases, they were found in 9 units: 7 with isolated cases, and 2 where clusters were identified (Table 2). All nosocomial cases of COVID-19 involving the patient and caregivers had cycle threshold (Ct) values below 33 and 27 respectively, which confirmed contagious status and recent acquisition. In the forensic medicine unit (social medicine, suicidology), the first case was community-acquired and identified on 16/03/2020. Starting with this case, 2 nosocomial cases were identified. The 3 patients had shared a room for about 2 days during each of their hospitalisations. The first nosocomial case appeared 3 days after the index case. After 6 days of hospitalization, fever was ascertained on 19/03/2020 and a nasopharyngeal swab on 20/03/2020 was positive for SARS-CoV-2. The second nosocomial case was hospitalised for 10 days, with the first symptoms appearing on 23/03/2020. A nasopharyngeal swab was sampled on 24/03/2020. On 02/04/2020, the unit received another patient, whose first symptoms were declared on 06/04/2020, and the case was considered as a community-acquired. The virus had probably been transmitted a roommate who was admitted on 25/03/2020 and presented a positive sample on 10/04/2020. All in all, 3 out of the 5 COVID-19 cases were nosocomial and none of the HCWs tested positive for SARS-CoV-2. In the Mood Disorder unit, the first case was considered as community-acquired insofar as he was hospitalised on 27/03/2020 and his first symptoms were declared on 29/03/2020. This case was linked to 2 nosocomial cases, the first of which had been hospitalised since 14/02/2020, while the first symptoms were declared on 03/04/2020. The second nosocomial case presented symptoms on the same day and had been hospitalised for about 3 months. Seven HCWs from this unit were tested for SARS-CoV-2, 4 of whom tested positive (57.1%).

**Table 1:** Nosocomial COVID-19 case characteristics

Characteristics	Nosocomial COVID-19 cases (n = 14)
Gender, n (%)	
Male	8 (57)
Median age in years (IQR)	63.7 (45.8–83.3)
Median time between hospitalization and symptom onset in days (IQR)	24.5 (11.5–42.0)
Patient 1	23
Patient 2	21
Patient 3	41
Patient 4	10
Patient 5	13
Patient 6	18
Patient 7	26
Patient 8	33
Patient 9	49
Patient 10	77
Patient 11	6
Patient 12	43
Patient 13	32
Patient 14	9
Risk factors, n (%)*	11 (78.6)
Overweight (25 < BMI** < 30)	2 (14.3)
Obesity (BMI** > 30)	0 (0.0)
Hypertension	6 (42.9)
Diabetes	4 (28.6)
Pulmonary disease	7 (50.0)
Cardiac Disease	5 (35.7)
Neuromuscular disease	1 (7.1)
Kidney Disease	2 (14.3)
Immunodeficiency	1 (7.1)
Co infection, n (%)	0 (0.0)
Intensive Care Unit stay, n (%)	1 (7.1)
Vital status, n (%)	
Dead	5 (35.7)

* Other than age > 65 years; ** BMI: Body Mass Index

**Table 2:** Distribution of nosocomial cases among units and number of healthcare workers (HCWs) tested

Unit	Nosocomial COVID-19 cases out of total COVID-19 cases in each unit	N of positive HCW out of total HCW sample*	Patient or HCW	Ct**	Acquisition
Neurology	1/1	0/3	Patient	33.48	HA
Geriatric	2/2	1/5	Patient	20.08	HA
			Patient	Missing data	HA
			HCW	27.5	–
Forensic Medicine (cluster 1)	3/5	0/3	Patient	29.13	CA
			Patient	Negative	CA
			Patient	Start of curve	HA
			Patient	25.85	HA
			Patient	31.65	HA
Pediatric surgery	1/1	2/5	Patient	33.49	HA
			HCW	18.97	–
			HCW	25.78	–
Thoracic oncology	2/2	0/1	Patient	12.52	HA
			Patient	15.66	HA
Endocrine Vascular Thoracic Surgery	1/1	1/4	Patient	20.46	HA
			HCW	23.63	–
Hepatology and Gastroenterology	1/3	0	Patient	18.15	HA
			Patient	29.45	CA
			Patient	Missing data	CA
Nephrology	1/1	0/3	Patient	16.65	HA
Mood disorder unit (cluster 2)	2/5	4/7	Patient	18.53	CA
			Patient	20.22	HA
			Patient	17.73	HA
			Patient	22.24	CA
			Patient	Negative	CA
			HCW	17.99	–
			HCW	23.52	–
			HCW	17.36	–
			HCW	20.47	–

*HCWs* healthcare workers, *Ct* cycle threshold. *HA* Hospital-acquired, *CA* Community-acquired

*Sampled between 5 and 15 days after the first symptoms of the first case

**The lowest Ct value between the 2 targets was chosen

**Fig. 1:**
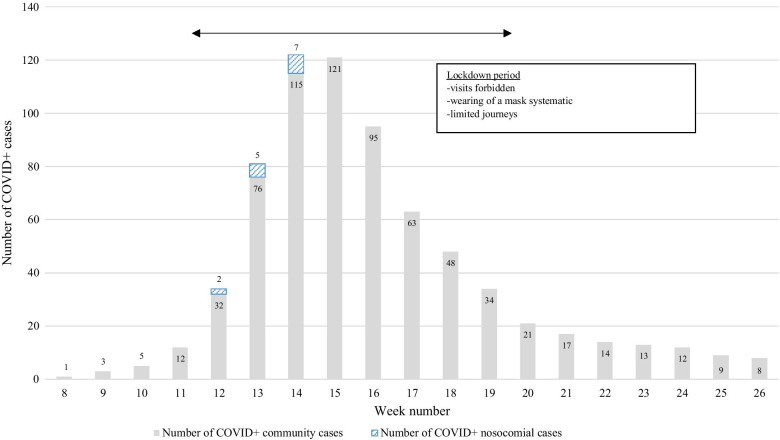
Time distribution of nosocomial-acquired cases among community-acquired cases of COVID-19

Figure 1 represents the distribution of nosocomial and community-acquired cases and underscores the fact that all the nosocomial cases were grouped in time and successive; they occurred in weeks 12, 13 and 14, the first 3 weeks of the lockdown.

As regards observation of hygiene measures (Table 3), the mask (FFP2 or surgical) was correctly worn by 94.7% of HCWs. FFP2 mask use was justified for 99.0% of HCWs. Concerning hand hygiene, 60.6% of HCWs carried it out when necessary, 15.5% achieved sufficiently long friction and 13.7% performed it correctly. Disinfection of shared equipment took place in 78.6% of observations. In 45.5% of observations, HCWs were too close to each other during breaks. Concerning hygiene measures for patients, wearing a mask when necessary (movement for technical exams or during care) was adequate among 81.5%. Physical distancing in common spaces was respected in 99% of observations. A screen was deployed in 76.2% of double occupancy rooms.

**Table 3:** Hygiene measures observed during audit in 88 units of Grenoble Alpes University Hospital

Hygiene measures observed	Compliance (%)
Wearing of mask (FFP2 or surgical) by HCWs	N = 693
Adequate	94.7%
Inadequate*	3.0%
Not wearing mask	2.3%
Wearing of FFP2 by HCWs	N = 693
Justified	99.0%
Hand hygiene	
Respect of indications	N = 449; 60.6%
Long enough friction	N = 277; 15.5%
Technique respected	N = 248; 13.7%
Disinfection of shared equipment	N = 98; 78.6%
Physical distancing respected for HCW (break rooms…)	N = 119; 54.6%
Wearing of mask for patients when needed	N = 260
Adequate	81.5%
Inadequate*	3.5%
Not wearing mask	15.0%
Screen in double occupancy rooms	N = 151
Correctly used (deployed)	76.2%
Physical distance respected between patients in common spaces (rehabilitation spaces, corridors…)	N = 73; 99.0%

^*^Meaning having the mask under the nose or the mouth

## Discussion

In this study carried out during the COVID-19 lockdown period in France, 5% of nosocomial cases were reported among all patients hospitalised for COVID-19 in our institution. Considering a range of incubation time up to 14 days, acquisition was questionable for 4 nosocomial cases with an onset of symptoms between 5 and 14 days. However, only 1 case occurred before 7 days of hospitalisation, therefore the number of nosocomial cases is probably not be excessively overestimated. Most of the nosocomial cases had risk factors for severe COVID-19 and 4 died from COVID-19. Survey of hygiene measures highlighted good compliance with mask wearing a but a lack of correct hand hygiene. During the first wave, the positive test rate in the Grenoble department (Isère) was lower than the mean of France, the the highest peak at the hospital being reached at week 13 with a positive rate of 18.17%.

Several studies have analysed the relative proportions of nosocomial COVID-19 cases. A meta-analysis conducted in Wuhan on nosocomial infection of COVID-19, SARS and MERS, showed a proportion of nosocomial COVID-19 at 44% [11]. Comparing our nosocomial rate to the literature is complex, due to the different definitions used in various studies. One study used the same definition of nosocomial cases in Japan and reported rate of nosocomial cases at 18.5% [12]. Another hospital in Spain, using an interval of 6 days, presented a nosocomial rate of 2.5% [13]. Percentages of nosocomial cases reported in 2 urology departments in Spain [14], a digestive surgery department in Paris [15], an orthopeadic surgery department in Spain [16] and a university Hospital in London [17] were 2.1%, 4.9%, 6.5% and 11.3% respectively. A study conducted in a large US Academic medical center found 2 nosocomial cases among 697 COVID-19 positive patients [18]. Finally, a study in three acute hospitals in Scotland found 19 (11%) nosocomial cases among 173 COVID-19 positive patients [19]. In comparison with influenza nosocomial cases, a meta-analysis [20] showed that the proportion of nosocomial cases among the total number of patients with influenza ranged from 15 to 59%. We can suggest two hypotheses to explain these differences and the low rate of COVID-19 nosocomial cases. First, the COVID-19 incidence rate in Grenoble during the first wave was rather low. However, other factors may also explain these results, including hygiene measures. During the COVID-19 outbreak, HCWs and patients systematically wore masks, which was not the case in the influenza epidemic. According to these results, universal wearing of a surgical mask by HCWs could be a way to control nosocomial transmission of respiratory viruses [21]. Another hypothesis concerns the lockdown and the ban on visits, which was never implemented during an influenza epidemic. Some studies have evaluated the effect of non-pharmaceutical interventions including the lockdown and proved their efficiency [22, 23]. In our study, all nosocomial cases were grouped within the first 3 weeks of lockdown. As fourteen days is the longest incubation time reported in the literature [24], we can suppose that the lockdown was efficient and helped to control nosocomial cases. It is important to note that given the high proportion of double occupancy rooms in our hospital for patients and a HCW staff shortage, quarantine of all exposed asymptomatic patients and HCWs was not possible, a factor that may have led to an increased number of nosocomial cases.

As regards hand hygiene we observed moderate compliance (60.6%) and, above all, poor technique (13.7%) and insufficient friction time (15.5%). A lack of time or a lack of knowledge could explain these results. Whatever the reason, education on the importance of hand hygiene is essential to promote these gestures. Moreover, the disinfection of shared equipment could be improved (78.6%). The survivability of Coronaviruses on inanimate surfaces has been shown to range from hours to days [25]. Even though the proportion of transmission from contaminated surfaces remains unknown, hand hygiene and disinfection of shared equipment are essential to avoidance of cross-transmission [26]. The Screen deployment in double occupancy rooms is another hygiene measure needing to be improved in our hospital. Indeed, in the first cluster, all the nosocomial cases were acquired in double occupancy rooms. Transmission from one patient to another sharing the same room, especially in SARS-CoV-2 with droplet transmission, is hard to control. This constitutes a huge challenge for our institution insofar as 65% of the rooms are double occupancy. Hygiene audits have shown a patient compliance rate of 81.5% for wearing a surgical mask when they moved or during care. In France, citizens are not used to wearing masks when they present respiratory symptoms or when they are in closed and crowded spaces; use of masks is generally limited to healthcare settings. In the second cluster, patient behavior may have influenced transmission. In the Mood Disorder unit, many manual activities shared between patients and HCWs such as card games or manual artistic activities are part of the treatment. Moreover, patients admitted in the 2 units with clusters are mainly psychiatric patients or patients with social difficulties. These units are at risk for cross-transmission because of major patient turnover, patients with difficulties complying with instructions, shared spaces and wandering patients. In our hospital, though there was no hand hygiene program targeting patients, but information on barrier measures was provided on screens in halls and patient rooms. Concerning the cluster where 7 HCWs were tested, 4 were infected. All patients and HCW were tested at the same time, so we cannot draw conclusions on the chronology of transmission [27]. Furthermore, physical proximity between HCWs during breaks could also be a way of transmission. A phylogenetic study based on sequencing could clarify these epidemiological links and confirm or not the existence of a single strain [28]. Of note, no cluster was identified in surgery units, probably due to the drastic reduction in their activity following the ban on elective surgery [29, 30].

The strengths of our study are based on prospective data collection. The same data have been collected from electronic medical records in our institution to follow nosocomial influenza cases, and we know this is a reliable and effective system [31]. Moreover, we were in touch with occupational health teams, infectious disease specialists and biologists in case of diagnosis uncertainty. All of these factors should help to reinforce knowledge of this emerging disease, especially concerning nosocomial cases, which are poorly described in the literature.

However this study has some limitations. First, our definition of nosocomial cases was based on 5-day incubation, whereas the incubation time described by the WHO is 1 to 14 days [24], meaning that we may have defined patients as nosocomial cases whereas they were not, or conversely. Given this wide range of incubation time, there is no optimum definition. We have chosen median incubation as a means of achieving early detection of potential nosocomial clusters in view of preventing further transmission, the objective being to avoid classifying a nosocomial case as community-acquired. Second, asymptomatic cases were not tested and systematic screening 14 days after discharge was not performed, certainly causing missed SARS-CoV-2 infections. Third, our institution was relatively spared and did not have as many cases of COVID-19 as other hospitals elsewhere. Finally, hygiene audits were performed after the lockdown period and did not adequately reflect the reality of lockdown period.

## Conclusions

In healthcare facilities, nosocomial transmission of respiratory viruses is a major issue and SARS-CoV-2 is not exempt from nosocomial transmission [32]. Hygiene measures, especially mask wearing and hand hygiene, are fundamental to control cross-transmission, and training of HCWs is an obviously necessary means of achieving good compliance. However, nosocomial transmission also depends on patient characteristics and their ability to comply with prevention measures. A specific and targeted policy should probably be established in units admitting patients who have difficulties observing these measures (psychiatry, social medicine or even geriatrics), the objective being to effectively prevent nosocomial transmission.

## Data Availability

The datasets used and/or analysed during the current study are available from the corresponding author on reasonable request.

## Abbreviations

CHU-GA: Grenoble Alpes University Hospital
COVID-19: Coronavirus disease 19
CT-scan: Computed tomographic scan
HCW: Healthcare workers
ICU: Intensive care units
IQR: Interquartile range
ORION: Outbreak Reports and Intervention studies of Nosocomial infection
RT-PCR: Reverse transcription polymerase chain reaction
SARS-CoV-2: Severe Acute Respiratory Syndrome coronavirus 2
WHO: World Health Organization
